# Placenta Percreta With Bladder Invasion: A Case Report Highlighting the Role of the Posterior Approach

**DOI:** 10.7759/cureus.81933

**Published:** 2025-04-09

**Authors:** Vijayanand Mani, Bhavyadeep Korrapati, Velmurugan Palaniyandi, Hariharasudhan Sekar, Sriram Krishnamoorthy

**Affiliations:** 1 Urology, Sri Ramachandra Institute of Higher Education and Research, Chennai, IND

**Keywords:** embolization, hysterectomy, partial cystectomy, placenta accreta, placenta percreta, posterior approach

## Abstract

Placenta accreta spectrum (PAS) represents a recently recognised continuum of abnormal placental invasion, including placenta accreta, increta and percreta, which is a rare, life-threatening complication of pregnancy affecting both mother and foetus, characterised by placental invasion beyond the serosa, often involving adjacent structures such as the urinary bladder. The increasing incidence is associated with rising caesarean section rates and other related factors such as advanced maternal age and infertility treatments. Surgical management remains challenging due to the risk of extensive bleeding, urological injuries and increased maternal morbidity. The posterior approach has emerged as a potential lifesaving technique in these complex cases. We present a series of three cases diagnosed with placenta percreta and bladder invasion, managed at a tertiary care centre. Preoperative imaging (ultrasound and magnetic resonance imaging {MRI}) confirmed abnormal placental adherence. All cases involved multidisciplinary approaches, including urologists, obstetricians and interventional radiologists. A posterior approach was employed to minimise blood loss, reduce bladder injury and improve surgical control. The first case describes a 26-year-old woman who underwent a posterior approach hysterectomy, which successfully reduced blood loss to 600 mL while preserving the bladder. The second case was a 30-year-old woman with grade 4 placenta previa and suspected percreta who underwent preoperative uterine artery embolisation (UAE), followed by a posterior approach hysterectomy and partial cystectomy. The third case involved a 35-year-old woman who developed a right ureteral injury requiring ureteric reimplantation with contralateral double-J stenting.

The posterior approach offers better vascular control, reduces bladder/ureteric injury and minimises blood loss. Early diagnosis, multidisciplinary planning and blood conservation strategies, such as uterine artery embolisation (UAE), are critical in improving outcomes. Future studies should further assess the long-term benefits of this approach in PAS management.

## Introduction

Placenta accreta spectrum (PAS) is a newly recognised spectrum of abnormal placental invasion, previously called the morbidly adherent placenta. It is a rare, life-threatening complication of pregnancy affecting both mother and foetus. PAS encompasses placenta accreta (beyond the endometrium), increta (up to the myometrium) and percreta (serosa and beyond) [1].

PAS occurs when the placenta grows too deeply into the uterus due to problems at the endometrial myometrial interface. If the inner layer of the myometrium is thinned out from the previous caesarean sections or curettage, the trophoblast tissue can invade into the serosa. This uncontrolled growth can extend into nearby organs such as the bowel and bladder [2].

Bladder involvement is often identified intraoperatively when placental detachment triggers severe haemorrhage [3]. Advanced techniques can reduce morbidity and blood loss, yet urologists are typically called in only during emergencies. Its surgical management presents a significant challenge, requiring multidisciplinary intervention involving obstetricians, urologists, interventional radiologists and anaesthetists. This case series highlights the lifesaving role of the posterior approach in managing placenta percreta with bladder invasion in three patients, emphasising surgical strategy, outcomes and lessons learned.

## Case presentation

Case 1

Modified Posterior Approach Reducing the Need for Massive Transfusion

**Figure 1:**
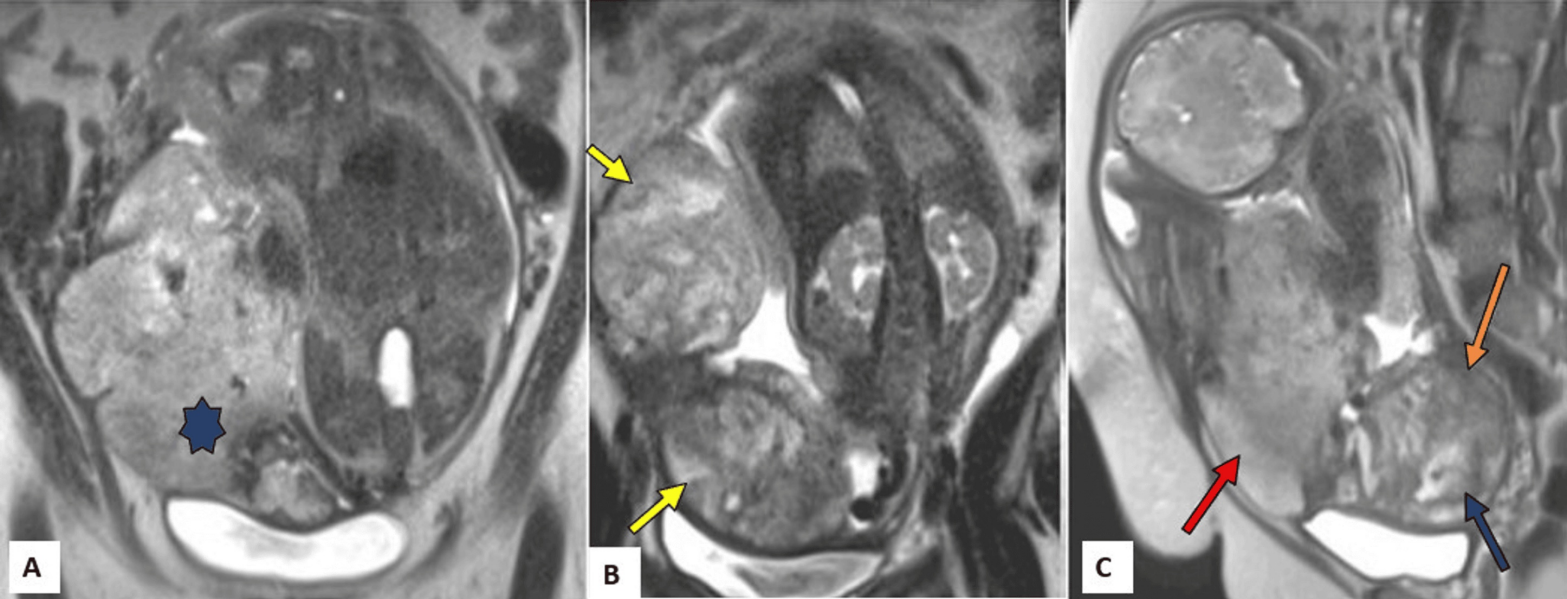
Magnetic resonance imaging of the pelvis with placenta: A and B, coronal sections; C, sagittal section. (A) Intrauterine foetal growth at 33 weeks with placentomegaly (blue star). (B) Focal uterine bulging areas with myometrium thinning along the lateral (upper yellow arrow) and anterior (lower yellow arrows) uterine walls. (C) Intraplacental haemorrhage (blue arrow) and retroplacental haemorrhage (orange arrow) along with uterine bulge in the anterior uterine wall (red arrow).

A 26-year-old woman, gravida 2, para 1 (G2P1), presented at 33 weeks of gestation with a prior history of hysterotomy at 26 weeks. During the third trimester, the patient was diagnosed with placenta percreta and bladder invasion based on magnetic resonance imaging (MRI) findings (Figure 1). She underwent an emergency lower segment caesarean section (LSCS) for foetal distress. The placenta vessels extended intraoperatively beyond the serosa into the dome of the bladder, pelvic wall and vagina. A classical caesarean section was performed on the right lateral wall, and a posterior approach was utilised for uterine and placental dissection with no ureteric involvement. The placenta was left in situ, and a total hysterectomy was performed one month later. The estimated blood loss was 600 mL, requiring one packed red blood cell (PRBC) unit. The patient had an uneventful recovery and was discharged on postoperative day (POD) 7 with no urological complications.

Case 2

Avoidance of Radical Bladder Resection

**Figure 2:**
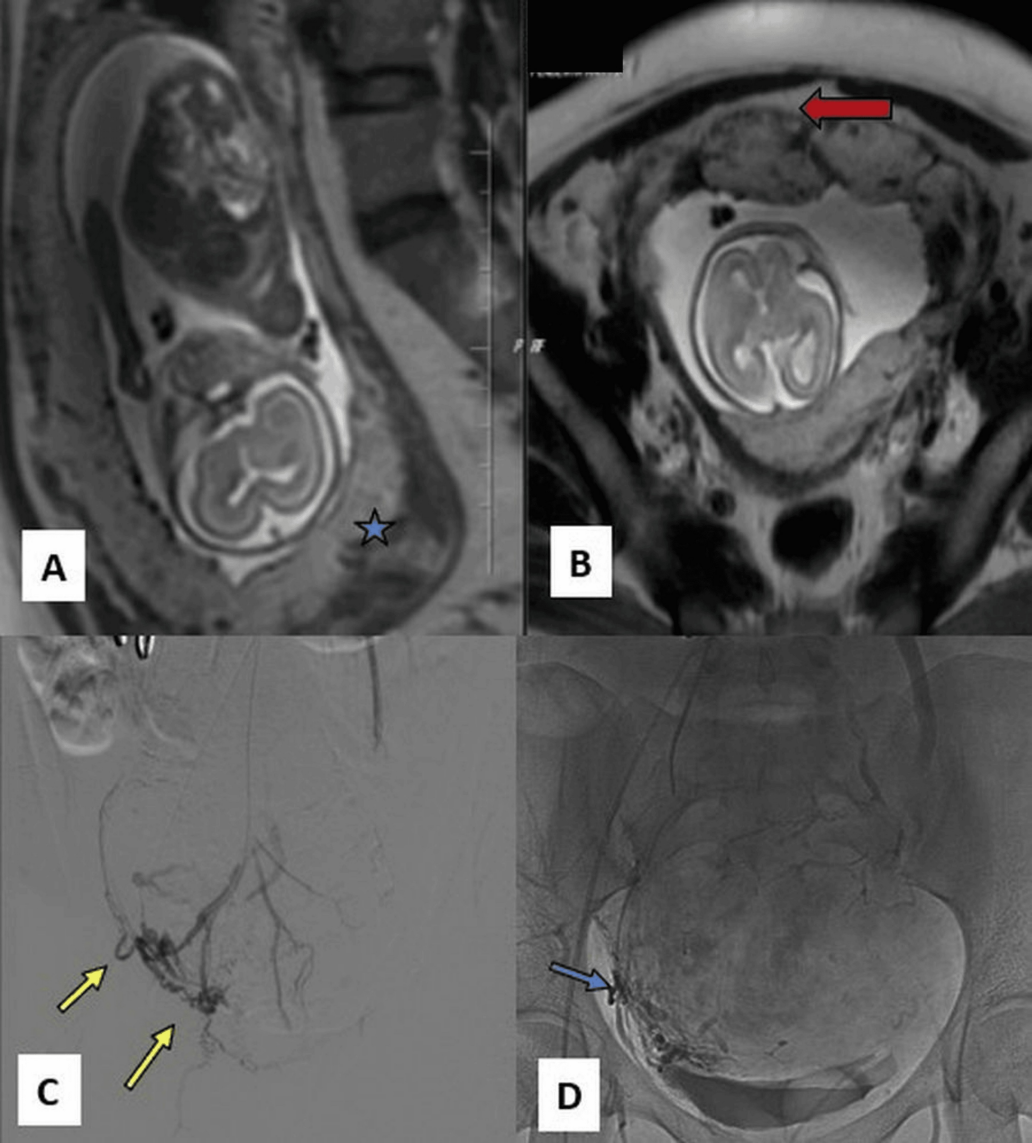
Magnetic resonance imaging of the pelvis with placenta and internal iliac angiogram: A, sagittal section; B, coronal section; C and D, right internal iliac angiogram. (A and B) Intrauterine foetal growth at 30 weeks with complete placenta previa and retroplacental haemorrhage. (A) A loss of interface between the decidua basalis and myometrium (blue star), with placental invasion into the serosa in the anterior layer (red arrow) (B). (C) Multiple tiny vesical feeders from the inferior and superior branches (yellow arrows) supplying the placenta percreta. (D) Post-embolisation images with an embolic cast within the placenta percreta branches (blue arrow).

A 30-year-old G2P1 woman at 34 weeks with a prior caesarean section presented with painless haematuria. MRI confirmed placenta percreta (Figure 2A, 2B). Cystoscopy showed no bladder involvement. She underwent an LSCS with perioperative uterine artery embolisation. Vessels encroached intraoperatively upon the uterine and bladder dome. Despite embolisation, vascularisation persisted via the vesical artery (Figure 2C, 2D). A posterior approach via the pararectal and presacral space enhanced visualisation, avoiding radical cystectomy. A partial cystectomy was performed, leaving the placenta in situ with a suprapubic catheter. The estimated blood loss was 2,500 mL. The patient received three units of PRBC and eight units of fresh frozen plasma (FFP). A suprapubic catheter was maintained for three weeks, and hysterectomy was completed three months later.

Case 3

Posterior Approach Preventing Recovering an Injured Ureter

**Figure 3:**
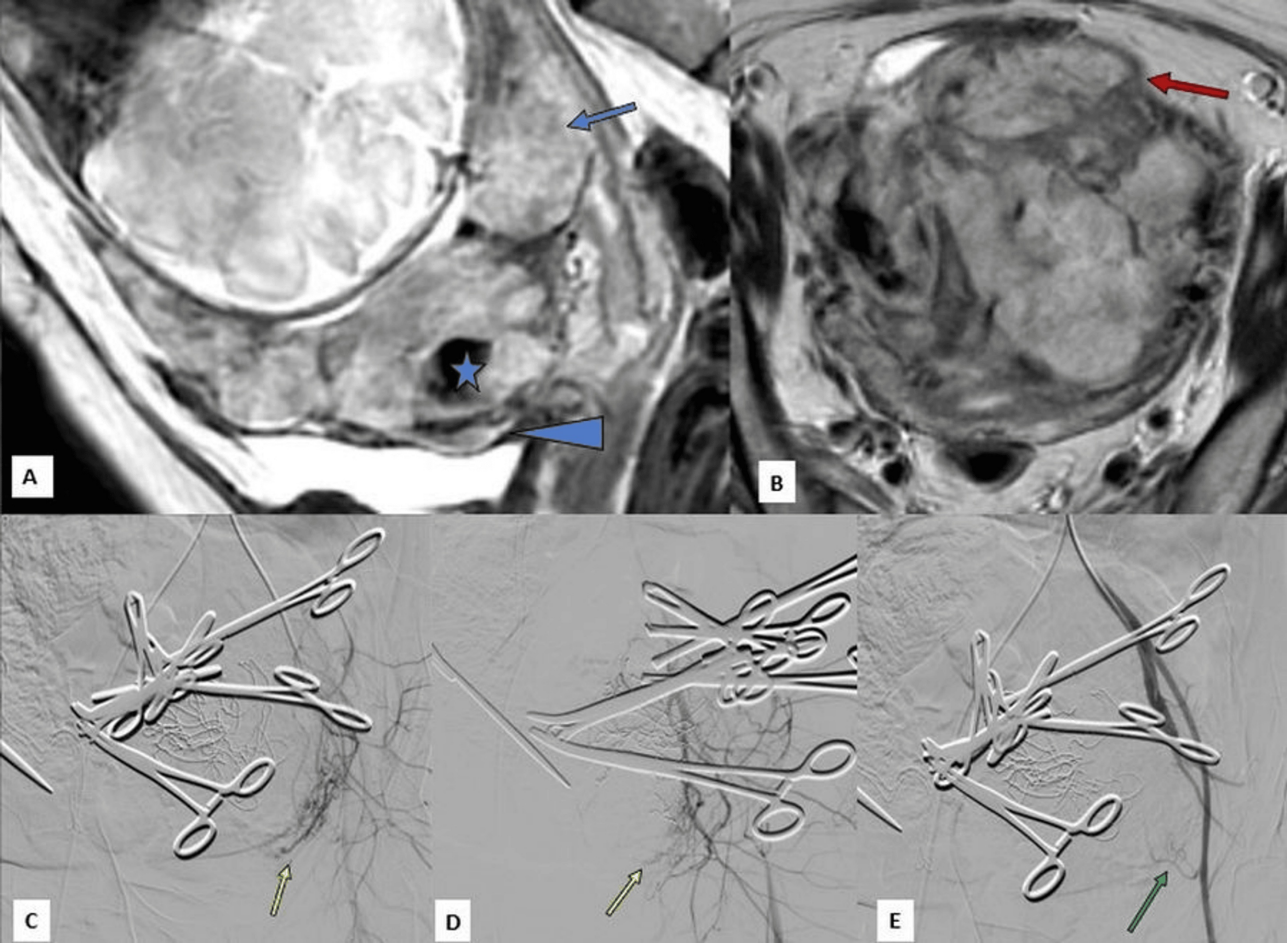
Magnetic resonance imaging of the pelvis with the placenta showing and internal iliac angiogram: A, sagittal section; B, coronal section; C-E, right internal iliac angiogram. (A, sagittal section) Grade 4 placenta previa (blue arrow) with intraplacental dark bands (blue star) and focal bulging of the anteroinferior uterine wall (blue arrowhead). (B, coronal section): The extension of the anteroinferior placenta up to the serosa and beyond (red arrow). (C-E) Internal iliac angiography, performed using a 5F Roberts uterine catheter (RUC), highlights inferior vesical arterial feeders (yellow arrow in C and D) supplying the placenta percreta. Post-embolisation angiography (E) confirms the complete obliteration of the abnormal vascular blush (green arrow).

A 35-year-old, gravida 4, para 2 (G4P2), woman at 33 weeks with two prior caesarean sections presented with haematuria and abdominal pain. MRI confirmed placenta percreta with bladder invasion (Figure 3A, 3B). A multidisciplinary surgical intervention was planned at 34 weeks. A midline vertical incision was made, and the foetus was delivered via upper segment hysterotomy. Extensive placental invasion into the bladder and bilateral ureters was confirmed. Despite aortic clamping, severe bleeding persisted, necessitating uterine artery embolisation (Figure 3C-3E). A right ureteric injury was identified intraoperatively. A posterior approach enabled controlled vascular ligation and precise dissection. A right ureteric reimplantation was performed, and a left double-J stent was placed. A total hysterectomy was done, sparing the bladder with partial cystectomy. The estimated blood loss was 2,300 mL, requiring four units of PRBC. The patient recovered well, was discharged on POD 8 and had catheter removal on POD 21. All three cases have been summarised in Table 1 for clarity and comparison.

**Table 1:** Summary of cases in our study MRI: magnetic resonance imaging

Case number	Summary	MRI finding	Type of intervention	Details of intervention	Outcomes	Take-home message
1	26/female, gravida 2, para 1, at 33 weeks, with previous hysterotomy	Placentomegaly, with intra- and retroplacental haemorrhage, with placental invasion beyond the serosa	Classical emergency lower segment caesarean section	Extensive infiltration of placenta percreta into the posterior bladder wall, vagina and bladder preserved by posterior approach with the retaining of placenta in situ	Preservation of the bladder and one unit of packed red blood cells transfused intraoperatively	Minimise blood loss in unplanned procedures and the preservation of bladder function
2	30/female, gravida 2, para 1, at 34 weeks, with previous caesarean section	Complete placenta previa with percreta	Elective lower segment caesarean section with uterine artery embolisation and partial cystectomy	Extensive placental invasion into the bladder dome. Uterine artery embolisation showing persistent feeder vessels from the superior and inferior vesical artery even after embolisation and vessels requiring partial cystectomy	Partial cystectomy, three units of packed red blood cells and four units of fresh frozen plasma transfused	Uterine artery embolisation is an adjuvant procedure to minimise blood loss; partial cystectomy can be employed to preserve bladder function
3	35/female, gravida 4, para 2, at 33 weeks, with two prior caesarean sections	Complete placenta previa with percreta	Elective lower segment caesarean section with uterine artery embolisation showing + aortic clamping, right ureteric reimplantation and left double-J stenting	Placental invasion into the posterior wall and involving the bilateral ureter	Right ureteric reimplantation, left double-J stenting and four units of packed red blood cells transfused	Ureteric and bladder injuries are expected complications and require meticulous detection to avoid it

## Discussion

The increasing incidence of placenta percreta is a major obstetric challenge worldwide, with a particularly notable rise in developing countries such as India. Advancements in assisted reproductive technologies and a rise in advanced maternal-age pregnancies have also contributed to these surges. Additionally, short interpregnancy intervals, multiparity and prior uterine surgeries increase the likelihood of placental invasion beyond the uterus, leading to complex surgical challenges [4].

The incidence of the spectrum has risen from one in 4,000 women (1970s) to one in 300 (2010s). An eightfold increase in three decades is due to a rise in caesarean sections [5]. Other associated risk factors include maternal age over 35 and procedures involved in the evaluation and management of infertility [5,6].

Usually, PAS involvement of the bladder is most often diagnosed during labour. Interestingly, gross haematuria is uncommon, occurring in only about one in four cases, even when bladder invasion is present [7].

Ultrasonography features include detecting placenta previa up to 80% [8]. Other sonographic features include the disruption of the normal hypoechoic zone between the myometrium and placenta, reduction in retroplacental myometrium thickness to <1 mm, multiple vascular lacunae within the placenta, irregularities at the uterine serosa-bladder interface and placental extension into the serosa and beyond. On colour flow, Doppler shows turbulent lacunar blood flow, sub-placental vascular network, discontinuities in myometrial blood flow and transforming vessels between the placenta and the uterine margin [9].

MRI findings associated with PAS include the existence of dark intraplacental bands on T2-weighted images, abnormal bump of the placenta or uterine contour, disruption of the uterine-placental interface and irregular or disorganised placental vasculature. MRI demonstrates a reasonably high diagnostic accuracy for predicting the placenta accreta spectrum, with a systematic review reporting a sensitivity of 70% and a specificity of 80% [10].

Cystoscopy frequently reveals either an ulcer or visible pulsating blood vessels on the posterior wall. However, the biopsy or fulguration of these lesions should be strictly avoided because such interventions can trigger severe haemorrhage [7,11].

Traditionally, an ‘anterior approach’ in a hysterectomy refers to a surgical technique where the surgeon accesses the uterus primarily through the anterior (front) part of the pelvic cavity, typically by first dissecting and then elevating the bladder to expose the vesicouterine pouch, which is the peritoneal reflection between the bladder and the uterus, allowing for the ligation of the uterine arteries and the subsequent removal of the uterus. This procedure is often used in a vaginal hysterectomy, where entering the anterior cul-de-sac is a crucial step to access the uterus properly [12].

Price et al. conducted ‘posterior approach hysterectomies’ on two women with suspected cases of placenta previa percreta [13]. In two instances, a conventional anterior approach for the mobilisation of the bladder leads to torrential bleeding and death.

Pelosi and Pelosi reported that the vesicouterine fold was approached via a broad ligament about 2 cm adjacent to the uterus at the level of the uterine artery, reaching the bladder from the lateral side [14]. Thus, the tunnel was created along the bladder curvature, and the placenta was separated from the bladder.

The modified retrograde approach by Hiroshi et al. from the pouch of Douglas posteriorly and gradually dissected circumferentially along the cervix facilitates the demarcation of the position of the lower uterine segment, bladder and invading placenta [15].

In all the approaches, there might be increased bleeding due to delay in the closure of the vaginal cuff; these can tack by holding the cervix technique by Matsubara et al. [16]. This method helps not only to control bleeding but also to delineate the cervix and vagina because the pregnancy cervix becomes soft and difficult to distinguish between the two structures.

The incidence of bladder or ureteric injury was noted in two-thirds of the patients; one required partial cystectomy, while the other required ureteric reimplantation with opposite-side double-J stenting. However, Tan et al. showed injury up to 90% (13 out of 14) among the placenta percreta patients, with one patient undergoing a nephrectomy for tacking the ureteric injury [17]. This may be due to the higher referral centre.

The advantages of the posterior approach include (a) better vascular control, as early devascularisation reduces blood loss [5]; (b) improved visualisation, since it allows access to the posterior uterus without disturbing the placental bed [16]; (c) reduced bladder injury, as it avoids direct anterior dissection; decreasing the risk of iatrogenic injury [14]; and (d) the preservation of bladder function [7]. Despite its advantages, the posterior approach presents particular challenges: (a) technical complexity, since the technique requires advanced surgical expertise and familiarity with retroperitoneal anatomy; (b) limited exposure in some cases, as severe adhesions or lateral placental extension may make posterior dissection difficult; and (c) the need for multidisciplinary involvement, because a well-coordinated surgical team is essential to manage potential vascular and urological complications effectively.

Learning points

This case series emphasises key learning points, focusing on the posterior approach to managing PAS. A posterior approach should be prioritised in cases with extensive bladder invasion because it helps reduce morbidity and complications. Early imaging and multidisciplinary planning remain essential for optimal outcomes, ensuring a comprehensive strategy for managing these complex cases. Finally, there is an urgent need for better screening guidelines, preemptive assessment strategies and multidisciplinary management approaches. Curtailing unnecessary caesarean sections, improving access to specialised maternal-foetal medicine services and ensuring timely surgical intervention can help mitigate the risks associated with placenta percreta and improve maternal outcomes.

The primary limitation of this study is that it is based on a limited number of cases, which restricts the generalisability of the findings. Additionally, the retrospective nature of the analysis limits the ability to establish causality or standardised recommendations. Furthermore, the absence of long-term follow-up prevents a comprehensive assessment of the patients’ urological and reproductive outcomes. Future larger, prospective studies are needed to validate the benefits and long-term implications of the posterior approach in managing the placenta accreta spectrum with bladder invasion.

## Conclusions

Placenta percreta presents significant challenges in obstetric and urological surgery. This case highlights the posterior approach as an effective technique for minimising blood loss, preserving bladder function and reducing surgical complications. A multidisciplinary approach, early diagnosis and preoperative planning are essential to improving patient outcomes. Further studies are needed to assess the long-term implications of this surgical strategy.
